# Predicting the new carbon nanocages, fullerynes: a DFT study

**DOI:** 10.1038/s41598-021-82142-2

**Published:** 2021-01-28

**Authors:** Mohammad Qasemnazhand, Farhad Khoeini, Farah Marsusi

**Affiliations:** 1 Department of Physics, University of Zanjan, P.O. Box 45195-313, Zanjan, Iran; 2 Department of Physics and Energy Engineering, Amirkabir University of Technology, P.O. Box 15875-4413, Tehran, Iran

**Keywords:** Chemistry, Energy science and technology, Materials science, Nanoscience and technology, Optics and photonics, Physics

## Abstract

In this study, based on density functional theory, we propose a new branch of pseudo-fullerenes which contain triple bonds with *sp* hybridization. We call these new nanostructures fullerynes, according to IUPAC. We present four samples with the chemical formula of C_4n_H_n_, and the structures derived from fulleranes. We compare the structural and electronic properties of these structures with those of two common fullerenes and fulleranes systems. The calculated electron affinities of the sampled fullerynes are negative, and much smaller than those of fullerenes, so they should be chemically more stable than fullerenes. Although fulleranes also exhibit higher chemical stability than fullerynes, but pentagon or hexagon of the fullerane structures cannot pass ions and molecules. Applications of fullerynes can be included in the storage of ions and gases at the nanoscale. On the other hand, they can also be used as cathode/anode electrodes in lithium-ion batteries.

## Introduction

Carbon is an element that has the potential to adapt to different molecular structures, and can form various molecular orbitals, such as *sp*, *sp*^2^, *sp*^3^, and so on. Diamond and graphite are the best-known bulk allotropes of carbon which their structures are made of *sp*^3^ and *sp*^2^ hybridization, respectively. Recently, cumulene and carbyne have been introduced as new carbon allotropes, having pure structures consisting of *sp* hybridization^1–7^. Some structures have more than one type of hybridization in their structures; for example, fullerene, which in addition to *sp*^2^ hybridization, has a slight hybridization of *sp*^3^, because of its curvature^8–11^. Graphyne is another example of new two-dimensional carbon materials, and unlike graphene, which includes *sp*^2^ hybridization, also includes *sp* hybridization^12–17^. It was shown that graphene can be transformed into fullerene cages^18^, now a question comes to mind: how would be the structures of the cages, if they are made of graphyne?

In this study, we introduce four new structures of carbon cages, which could be new branches of the pseudo-fullerenes family. The geometry of fullerene consists of twelve pentagon rings and a variable number of hexagons. If the structure of the fullerenes is saturated with hydrogen, the orbital hybridization of the bonds shifts from *sp*^2^ to *sp*^3^ and is called fullerane^19^.

In this work, we explore a new class of fullerenes derivatives by adding *sp* orbital hybridization to fulleranes structures. Then, by comparing the structural, electrochemical, and optoelectrical properties of these structures with fullerenes and fulleranes, we show that this new class of carbon cages does not belong to the category of fullerenes and fulleranes. It is best to call them fullerynes, in the style of IUPAC, and due to the triple bonds with *sp* hybridization orbitals in their structures. We show that the fullerynes may have interesting applications in the field of nanotechnology, including hydrogen storage nanocapsules and cathode/anode electrodes in lithium-ion batteries^20,21^.

## Results

**Figure 1:**
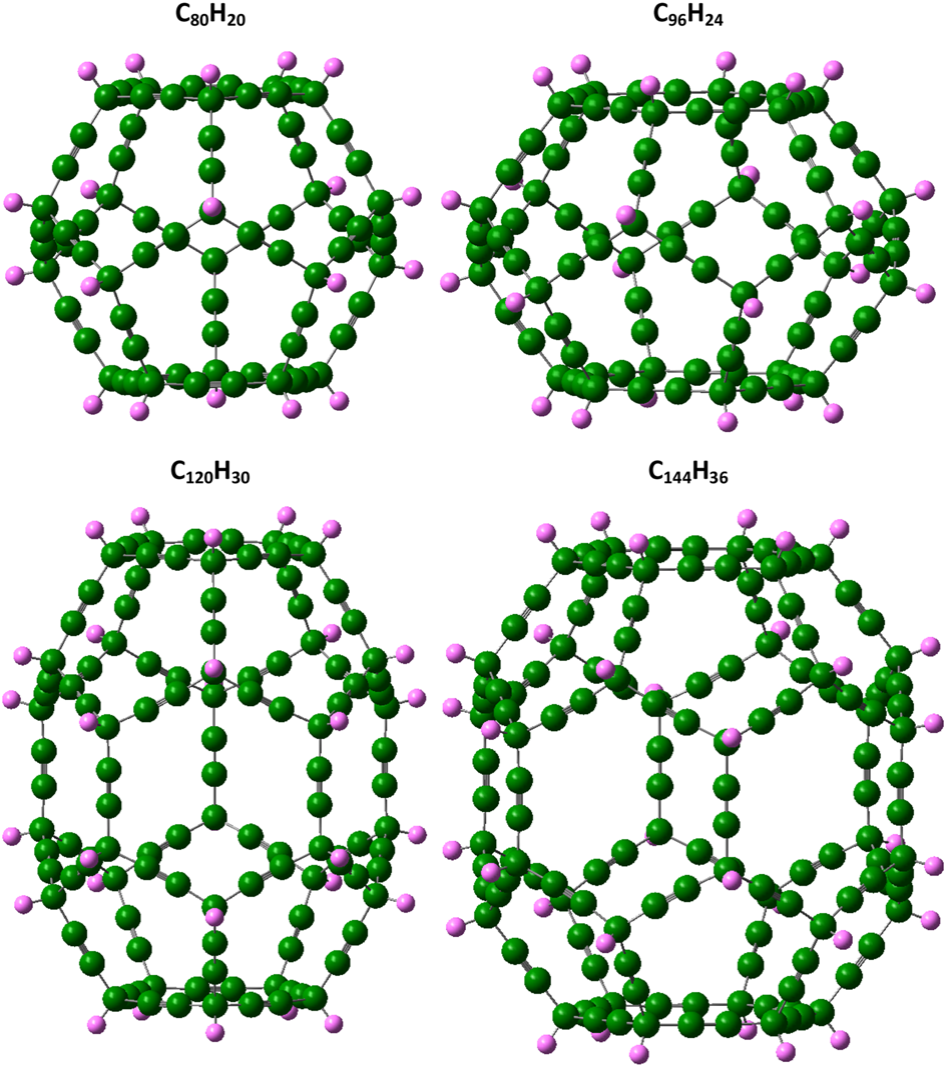
The fullerynes, C_80_H_20_, C_96_H_24_, C_120_H_30_, and C_144_H_36_ structures, were obtained, respectively, for C_20_H_20_, C_24_H_24_, C_30_H_30_ and C_36_H_36_ fulleranes structures.

Fulleryne, a new carbon cage, is formed by adding two carbon atoms to each edge of the fullerane. So it can be concluded that in pure fullerynes, twice the number of edges is added to the carbon number of each structure. In this study, the corresponding C_80_H_20_, C_96_H_24_, C_120_H_30_, and C_144_H_36_ fulleryne structures were obtained, respectively, for C_20_H_20_, C_24_H_24_, C_30_H_30_ and C_36_H_36_ fulleranes structures. In general, each fullerane with a chemical formula C_n_H_n_ has a fulleryne corresponding to the formula C_4n_H_n_. The carbon skeleton of each fulleryne structure is shown in Fig. 1.

It should be noted that by the method, we achieved the enthalpy of formation of the fullerene, C_60_, about 2580 kJ/mol, besides, we obtained heat of formation about 7900 kJ/mol for fulleryne C_80_H_20_. These structures are stable although they have high energy levels. Because, they do not have any imaginary frequencies, in the vibrations of their structure, so with this vibrational spectrum, we can conclude that the structures located at the minimum potential energy surface.

**Figure 2:**
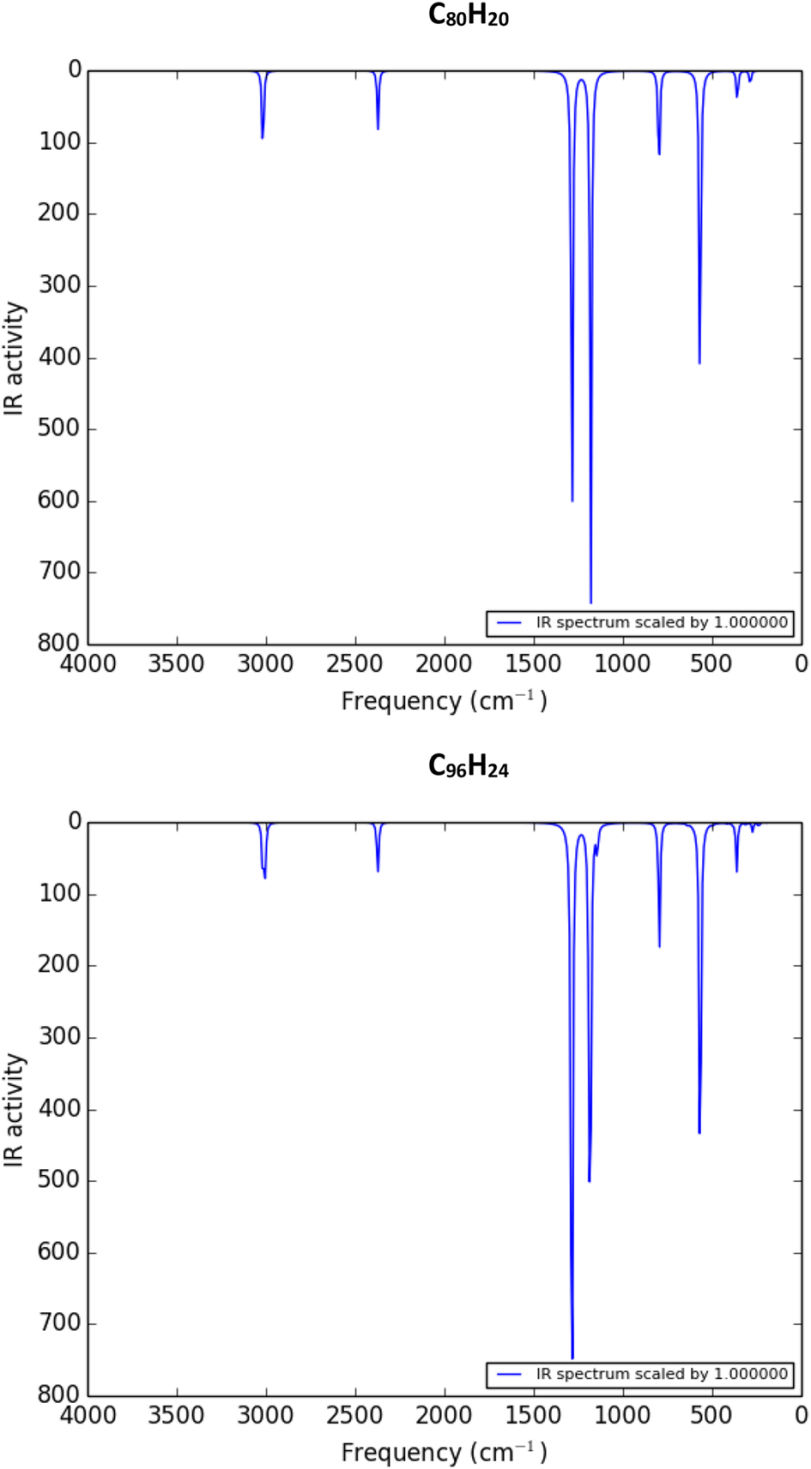
The infrared spectra for the fullerynes, C_80_H_20_ and C_96_H_24_.

**Figure 3:**
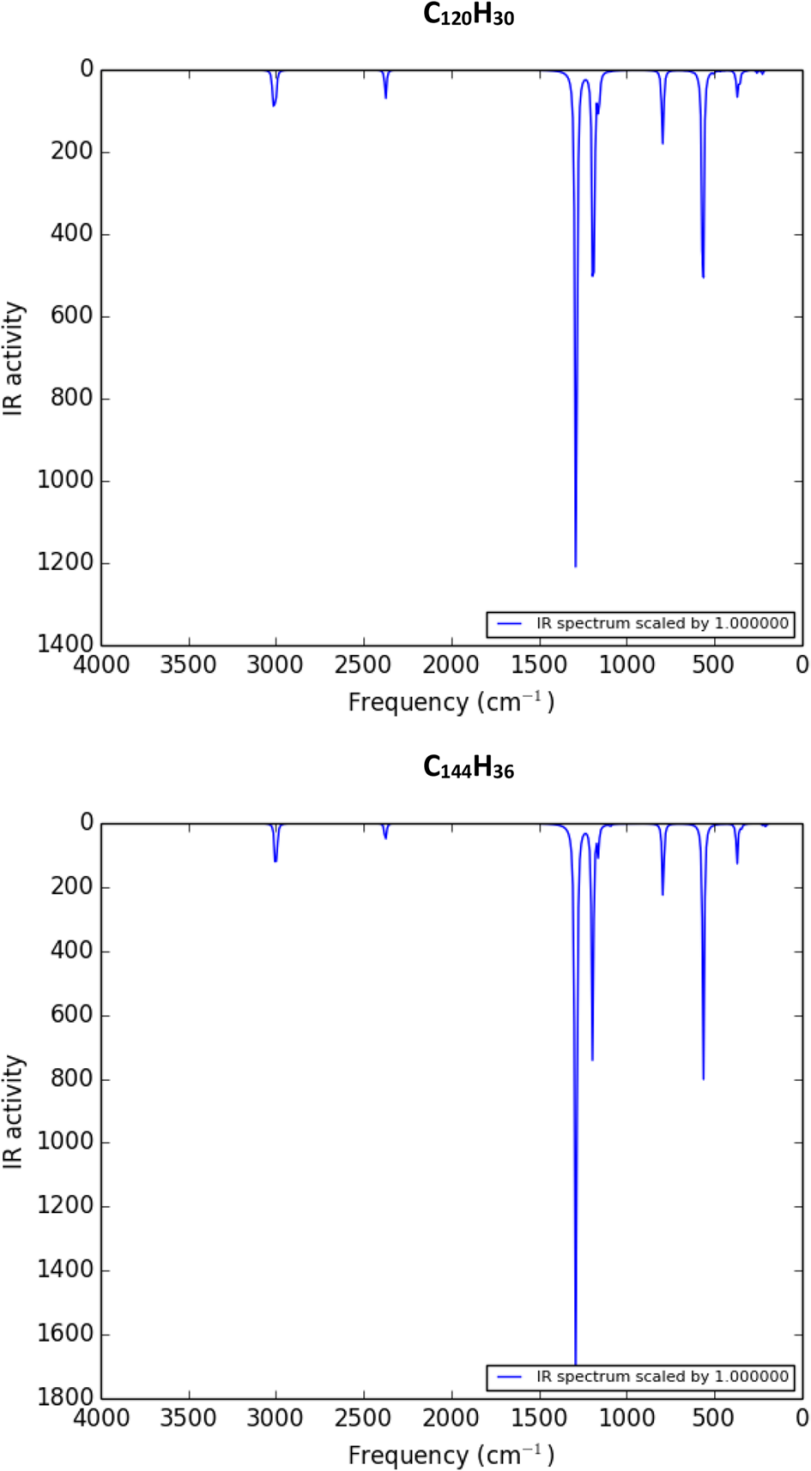
The infrared spectra for the fullerynes, C_120_H_30_ and C_144_H_36_.

We use the following formula to compare the stability of a fulleryne cage with a fullerene cage consisting of the same number of carbon atoms^22,23^.

1 $$\Delta E=\frac{4n}{60}E\left(F\right)+\frac{n}{2}E\left({H}_{2}\right)-{E(C}_{4n}{H}_{n}),$$

where *E*(*F*), *E*(*H*_2_) and *E*(*C*_4*n*_*H*_*n*_) stand for the ground-state total energies of a fullerene cage, a single hydrogen molecule, and a fulleryne cage with the chemical formula of *C*_4*n*_*H*_*n*_, respectively. The results show that fullerene cages are more stable than fullerynes (of about 500 meV per each carbon atom). However, fulleryne structures show significant binding energies, *ΔE*_*B*_, which are comparable to *ΔE*_*B*_ for fullerene cages. *ΔE*_*B*_ for fulleryne cages, *C*_*4n*_*H*_*n,*_ are calculated using equation below:

2 $$\Delta {E}_{B}=\left[4n E\left(C\right)+\frac{n}{2}E\left({H}_{2}\right)-E\left({C}_{4n}{H}_{n}\right)\right],$$

where *E*(*C*) is the ground-state total energy of a single carbon atom. On the other hand, the calculated frequencies of the vibrational modes do not show any negative frequencies, so the structures are stable^24^. The corresponding infrared spectra are presented in Figs. 2 and 3.

By considering the above figure, we can understand the vibrational properties of the introduced fullerynes. In the infrared spectrum diagrams of the introduced fullerynes, the functional regions can be classified into three regions: first, the region of less than 500 cm^−1^ is related to rocking and wagging vibrations of *sp*^3^ carbon bonds to the fullerynes vertices, and bending vibrations of the acetylene functional groups created by adding two carbons at the edges. Second, the region between 500 and 1000 cm^−1^ related to scissoring vibrations of the *sp*^3^ carbon bonds and twisting vibrations of the acetylene functional groups. Third, in the area between 1000 and 1500 cm^−1^, rocking and wagging vibrations occur in the C–H bonds and stretching vibrations in the C–C bonds.

But in fingerprint regions in the infrared spectrum of the introduced fullerynes, there are only two obvious troughs, the first related to the stretching vibrations in the acetylene functional groups, and the second to the stretching vibrations in the C–H bonds.

**Figure 4:**
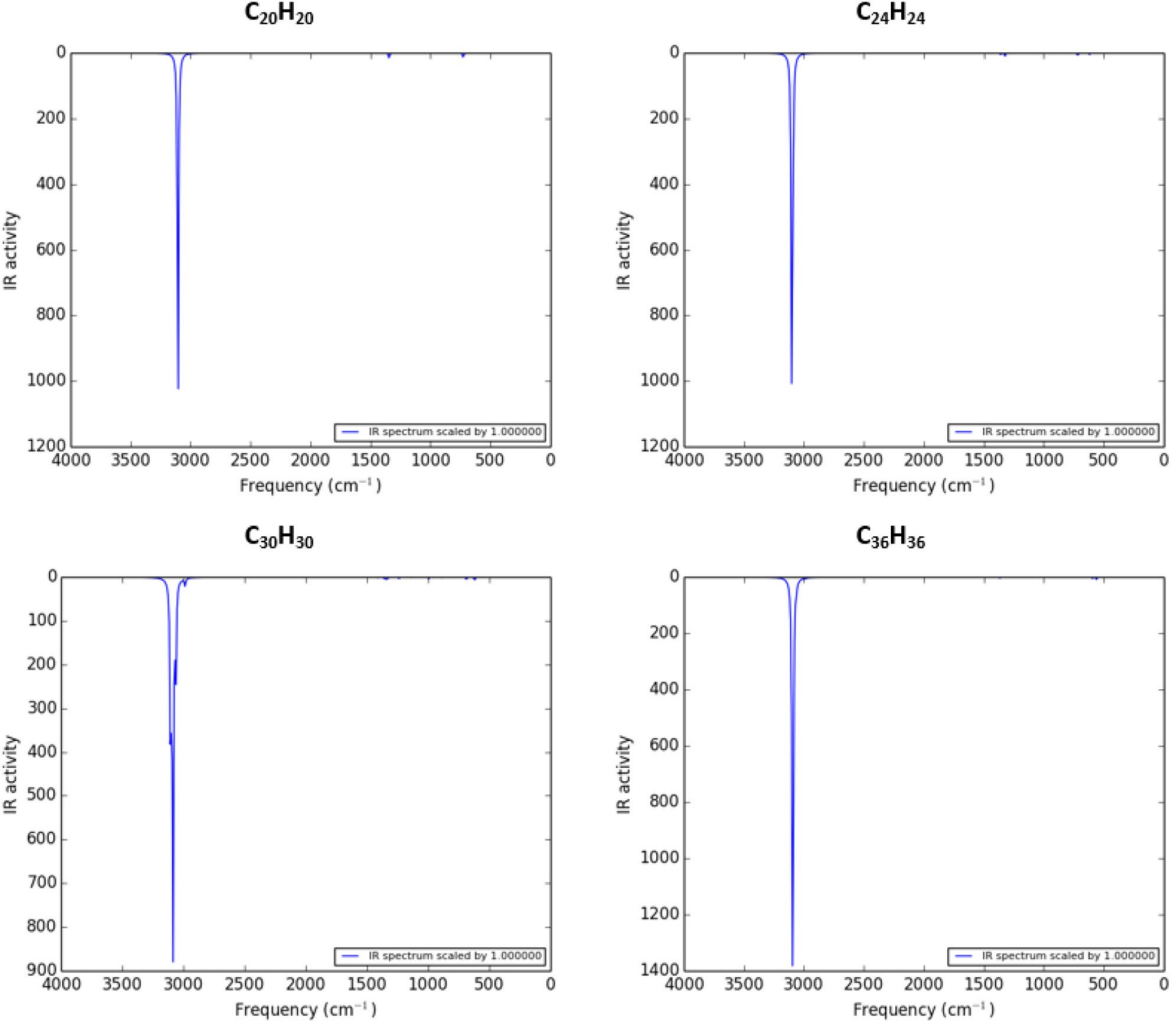
The infrared spectrum of fulleranes, foundations for introducing new structures.

At the end of this section, Fig. 4 shows the infrared spectra of fullerane structures, foundations to introduce fullerynes.

After determining the stability of the fulleryne structures, we present fullerynes properties in the following three parts: the structural, electrochemical and optoelectrical properties.

### Structural properties

To describe the dimensions of the structures investigated in this study, it is assumed that each is in a suitable box, and the dimensions of that hypothetical box were obtained using a graphical interface, the GaussView software^25^.

**Figure 5:**
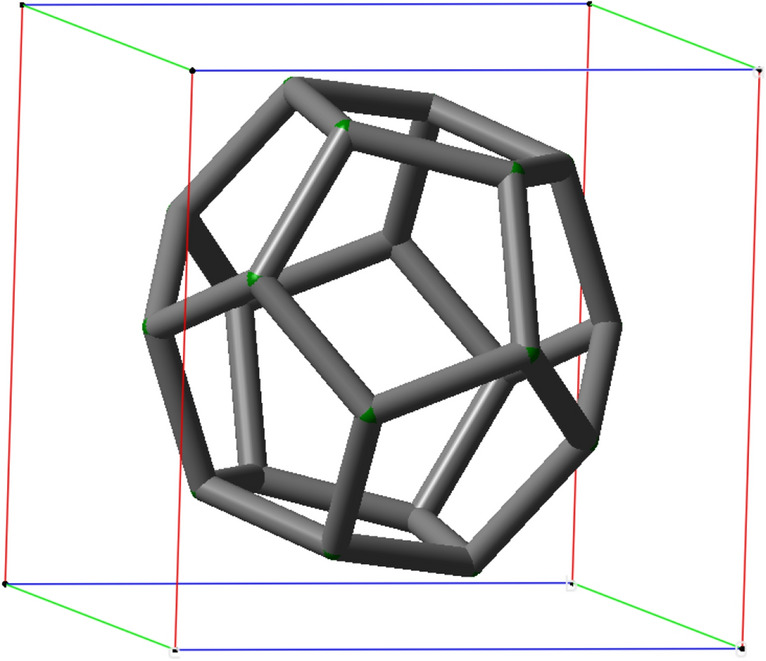
The C_20_ structure inside a hypothetical box introduced by GaussView software. To describe the dimensions of the structures investigated in this study, we assumed that each is in a suitable box. It helps us to approximate the diameter of each structure**.**

Then the size of this box helps us to approximate the diameter of each structure. An example of a hypothetical box for the structure of C_20_ is given in Fig. 5.

**Figure 6:**
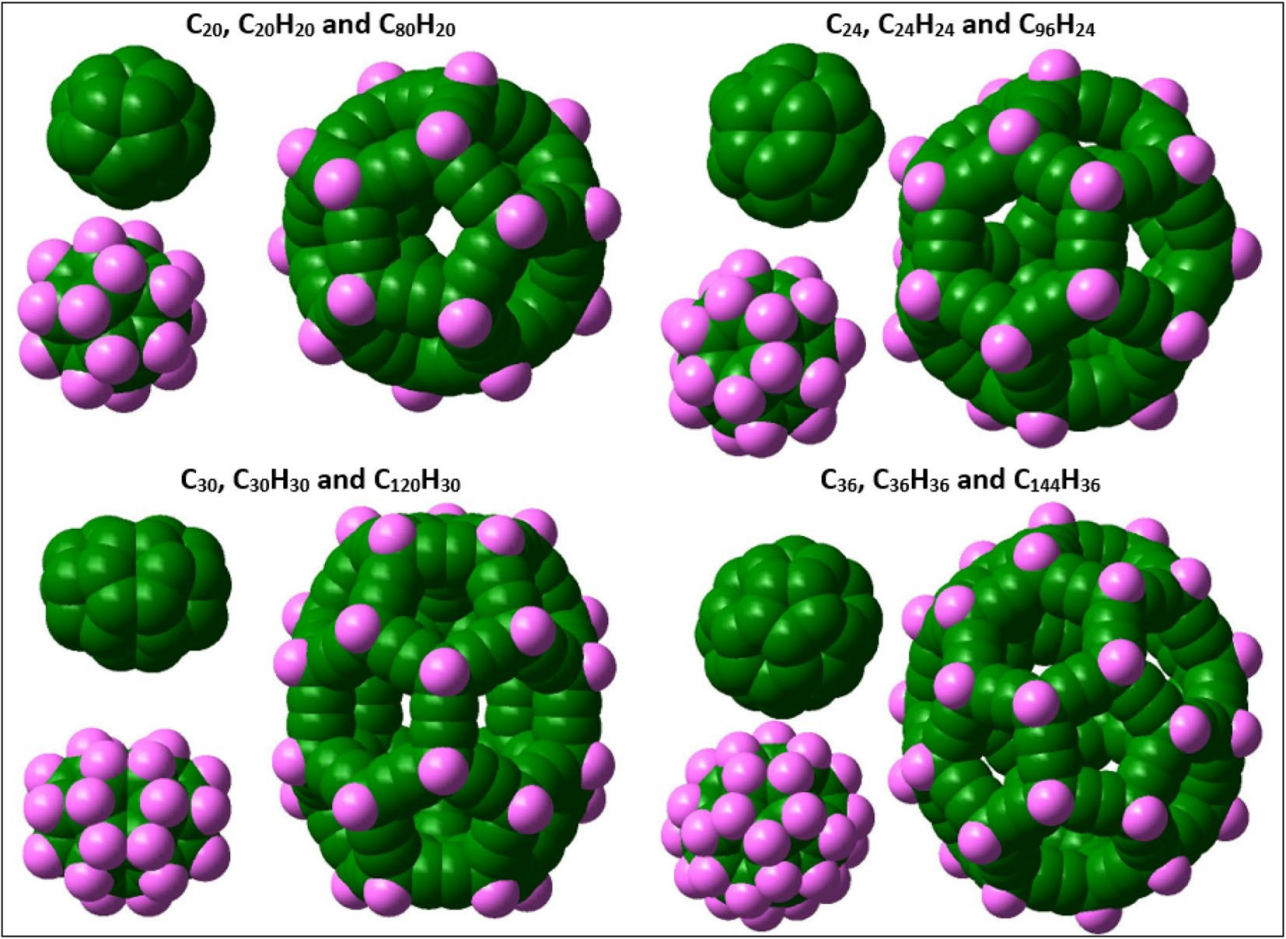
For better visualization and comparison of fullerenes (C_n_), fulleranes (C_n_H_n_) and fullerynes (C_4n_H_n_) with each other, the graphic models are presented. The symmetry of the fullerane and fulleryne structures is higher than the symmetry of the fullerene structures, and ignoring about 0.3 Å, the changes in the size of the fullerenes can be classified into symmetrical groups of fulleranes and corresponding fullerynes.

The size of the hypothetical box, along with the symmetry of each structure is given in Table 1. To imagine and compare the size of the introduced fulleryne structures, the dimensional characteristics of the corresponding fullerane and fullerene structures are given as follows in Fig. 6.

**Table 1:** Geometrical properties of the introduced fullerynes and the corresponding fulleranes and fullerenes (in units Å and eV).

Cages	SYM	BOX (a, b, c)			E_tot_	BE
C_80_H_20_	Ih	14.6	14.6	14.4	− 83,206.76	8.47
C_96_H_24_	D6d	16.7	16.7	11.8	− 99,848.07	5.96
C_120_H_30_	D5h	18.0	15.1	14.8	− 124,810.09	5.11
C_144_H_36_	D6h	17.8	17.7	16.6	− 149,772.02	5.11
C_20_H_20_	Ih	7.5	7.5	6.7	− 21,062.94	11.35
C_24_H_24_	D6d	8.1	8.1	6.2	− 25,274.61	11.30
C_30_H_30_	D5h	8.4	7.7	7.5	− 31,592.11	11.27
C_36_H_36_	D6h	8.7	8.4	8.1	− 37,908.18	11.20
C_20_	Ih	5.0	5.0	4.9	− 20,714.29	7.49
C_24_	D6d	5.8	5.8	4.1	− 24,859.11	7.57
C_30_	D5h	6.5	5.1	5.1	− 31,080.30	7.79
C_36_	D6h	6.3	6.0	5.8	− 37,305.00	8.03

**Figure 7:**
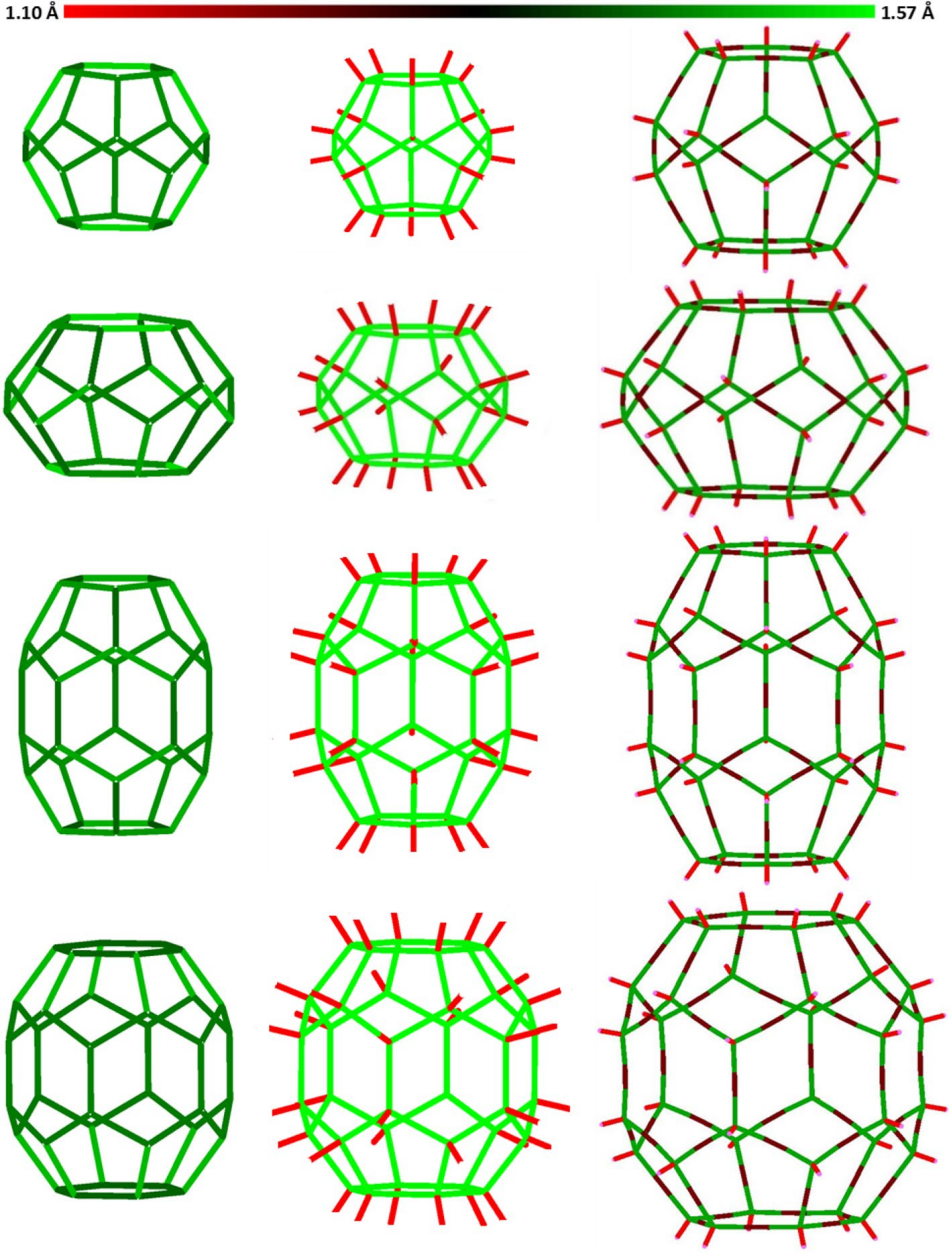
Carbon–carbon bond length variations, respectively, in the three structures of fullerenes, fulleranes, and fullerynes. The range of bond length changes starting from the lowest value, 1.10 Å, which corresponds to the length of the carbon–hydrogen bond in fulleranes and fullerynes, until the maximum value, 1.57 Å, related to carbon–carbon bonding in fulleranes. The lengths of the single and triple bonds of the investigated fullerynes are 1.49 and 1.22 Å, respectively.

The first column in Table 1 shows the type of structural symmetry. It can be seen from the above table that the symmetry of the corresponding structures is the same. However, the symmetry of the fullerane and fulleryne structures is higher than the symmetry of the fullerene structures, and ignoring about 0.3 Å, the changes in the size of the fullerenes can be classified into symmetrical groups of fulleranes and corresponding fullerynes. To illustrate this, it is necessary to carefully examine the geometric properties of each structure. However, before that, for better visualization and comparison of these three types of carbon cages, the corresponding structures are shown in Fig. 7.

The introduced fullerynes in this study, have two types of carbon bonds in their structures: the first type of bond is the bond between the vertex carbons and the edge carbons, and the other is the carbon bonds located at the edges of the fulleryne structures, and this is a triple bond^26^. The lengths of the single and triple bonds of the investigated fullerynes are 1.49 and 1.22 Å, respectively. In fulleranes with the same symmetry group as fullerynes, the length of all carbon–carbon bonds is about 1.57 Å, while in fullerenes, the bond lengths are not uniform like those of fulleranes, because of the resonance that makes the fourth electron^27^. For this reason, they are not symmetrical at the level of the fulleranes and the fullerynes, and with some slight exaggeration, they can fit into their symmetrical group. Using the colors, the bond length changes for each of the fullerenes, fulleranes, and fullerynes are schematically illustrated in Fig. 7.

The range of bond length changes starting from the lowest value of 1.10 Å, which corresponds to the length of the carbon–hydrogen bond in fulleranes and fullerynes, and it continues until the maximum, 1.57 Å, related to carbon–carbon bonding in fulleranes. The red indicates the shortest bond length, the darker the middle bond length, and the light green the longer bond length.

Now we want to examine the stability of the cages. The total energy of each nanoparticle is related to the number of atoms forming it, so we can’t use it as a factor for comparison, and use the binding energy, because it determines the contribution of a particle to the stability of the structure. We use the following equation to calculate the binding energy^28^:

3 $$BE=\frac{{(E}_{cage}-{nE}_{atom})}{n}.$$

Considering the binding energy with the nanocages, one can see that we are facing three different families of nanoparticles. Fulleranes with a binding energy of about 11 eV could be the most stable family of nanocages under study. On the other hand, the binding energy of the two groups of fullerenes and fullerynes is closer together and less than fulleranes. However, the fullerynes cannot be from the fullerenes family because the binding energy of the fullerynes, unlike the fullerenes, decreases as the nanocages grow larger, and in this case, it is similar to the fulleranes.

### Electrochemical properties

Electrochemical properties are another factor that can be used to classify nanoparticles. The following table provides information on the energy levels of the HOMO and LUMO orbitals, the size of the HOMO–LUMO gap, and finally, chemical potential (µ); in other words, it is the Fermi energy level^29,30^.

4 $$\upmu =\frac{({E}_{HOMO}+{E}_{LUMO})}{2}.$$

We calculate the electronegativity (χ), too, from the average value in ionization potential and electron affinity. The following equations can be used to estimate the values of ionization energy and electron affinity^28^:

5 $$IP=E\left(neutral\right)-E\left(kation\right).$$

6 $$EA=E\left(neutral\right)-E\left(anion\right).$$

We used the following relationships to obtain the chemical potential, the chemical hardness, and global softness:

7 $$\upeta =\frac{({E}_{HOMO}-{E}_{LUMO})}{2}.$$

8 $$\upsigma =\frac{1}{\upeta }.$$

Now, with the help of the above equations, we calculate electrophilicity for different structures with the following relation^31,32^:

9 $$\upomega =\frac{{\upmu }^{2}}{2\upeta },$$

where it is a newer index and more accurately distinguishes between structures. The electronic properties of the fullerynes introduced in this study are presented in Table 2. The following table also lists the electronic properties of corresponding fulleranes and fullerenes to illustrate the difference of fullerynes with them.

**Table 2:** Electronic properties of the introduced fullerynes and the corresponding fulleranes and fullerenes (in units eV).

	HOMO	LUMO	H–L	µ	IP	EA	χ	η	σ	ω
**Fullerynes**										
C_80_H_20_	− 7.07	0.15	7.22	− 3.46	8.15	− 1.03	3.46	3.66	0.27	1.66
C_96_H_24_	− 7.00	0.13	7.13	− 3.43	7.86	− 0.94	3.43	3.56	0.28	1.65
C_120_H_30_	− 6.95	0.18	7.13	− 3.38	7.84	− 0.9	3.38	3.56	0.28	1.60
C_144_H_36_	− 6.90	0.18	7.08	− 3.36	7.60	− 0.83	3.36	3.54	0.28	1.59
**Fulleranes**										
C_20_H_20_	− 7.08	2.01	9.09	− 2.54	8.68	− 3.43	2.54	4.54	0.22	0.71
C_24_H_24_	− 6.99	1.73	8.72	− 2.63	8.50	− 3.1	2.63	4.36	0.23	0.79
C_30_H_30_	− 6.60	1.43	8.03	− 2.58	8.00	− 2.73	2.58	4.02	0.25	0.83
C_36_H_36_	− 6.48	1.09	7.57	− 2.69	7.79	− 2.33	2.69	3.78	0.26	0.96
**Fullerenes**										
C_20_	− 5.90	− 4.14	1.76	− 5.02	7.64	2.45	5.02	0.88	1.14	14.32
C_24_	− 5.96	− 4.76	1.20	− 5.36	7.60	3.14	5.36	0.60	1.67	23.94
C_30_	− 5.92	− 4.66	1.26	− 5.29	7.40	3.17	5.29	0.63	1.59	22.21
C_36_	− 5.97	− 4.97	1.00	− 5.47	7.38	3.55	5.47	0.50	2.00	29.92

The above data clearly shows that we have three different spectra of nanostructures. The most chemical reactivity of the above structures are fullerenes, which have more electron electronegativity, chemical softness, and electrophilicity than fulleranes and fullerynes. As the size of the fullerenes increases, their reactivity increases too. On the other hand, fulleranes have the least reactivity, like the fullerenes, as their size increases, their reactivity grows.

**Figure 8:**
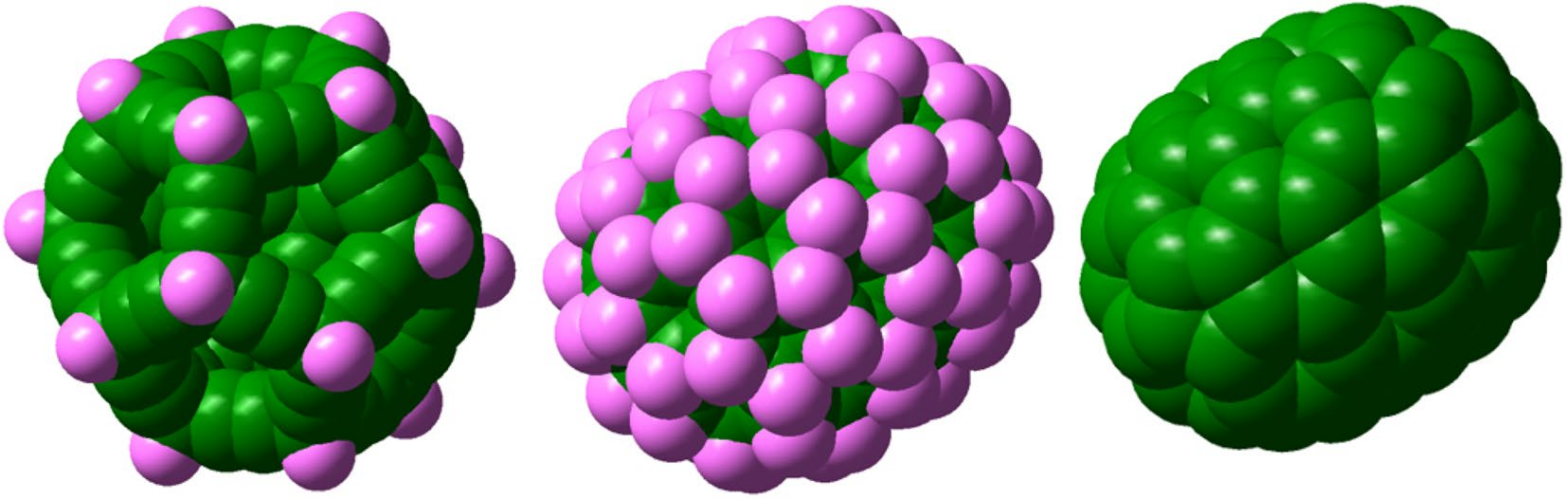
Three carbon nanocages made of 80 carbons: fulleryne, C_80_H_20_, fullerane, C_80_H_80_ and fullerene, C_80_.

It is necessary to note that so far, we only compared structures with the same symmetry, but fullerynes have more carbon atoms than those of fulleranes and fullerenes. Obviously, it is not suitable to compare their stability or reactivity. Therefore, in completing the analogy made in the research, we compared three carbon nanocages made of 80 carbons, the C_80_H_20_ fulleryne, C_80_H_80_ fullerane and C_80_ fullerene. Figure 8. shows the models of the three nanocages mentioned.

The electronic properties of the three cages are given in Table 3. We compare their electronic properties. Finally, with the help of the electrophilicity index, it becomes clear that these three cages are each of different categories.

**Table 3:** Electronic properties of three carbon nanocages made of 80 carbons: fulleryne, C_80_H_20_, fullerane, C_80_H_80_ and fullerene, C_80_ (in units eV).

Cages	HOMO	LUMO	H–L	*µ*	*IP*	*EA*	*χ*	*η*	*σ*	*ω*
C_80_H_20_	− 7.11	− 0.30	6.83	− 3.69	8.15	− 1.03	3.69	3.41	0.29	2.00
C_80_H_80_	− 5.64	0.96	6.59	− 2.34	6.66	− 1.89	2.34	3.30	0.30	0.83
C_80_	− 5.55	− 4.60	0.94	− 5.08	6.62	3.54	5.08	0.47	2.12	27.3

The fullerenes are the same fulleranes that have *sp* hybridization added to their structure. Since the energy level of *sp* orbitals is lower than that of *sp*^3^ orbitals, so the HOMO of fulleryne structures will not be much different from HOMO of fullerane structures. But on the other hand, the energy level of anti-bonding orbital of *sp* is lower than anti-bonding orbital of *sp*^3^. Therefore, the energy level of the LUMO orbital in fulleryne structures is lower than that of fullerane structures. The lower LUMO orbital, caused the higher electron affinity. So the fulleryne electron affinity is more than fullerane.

**Figure 9:**
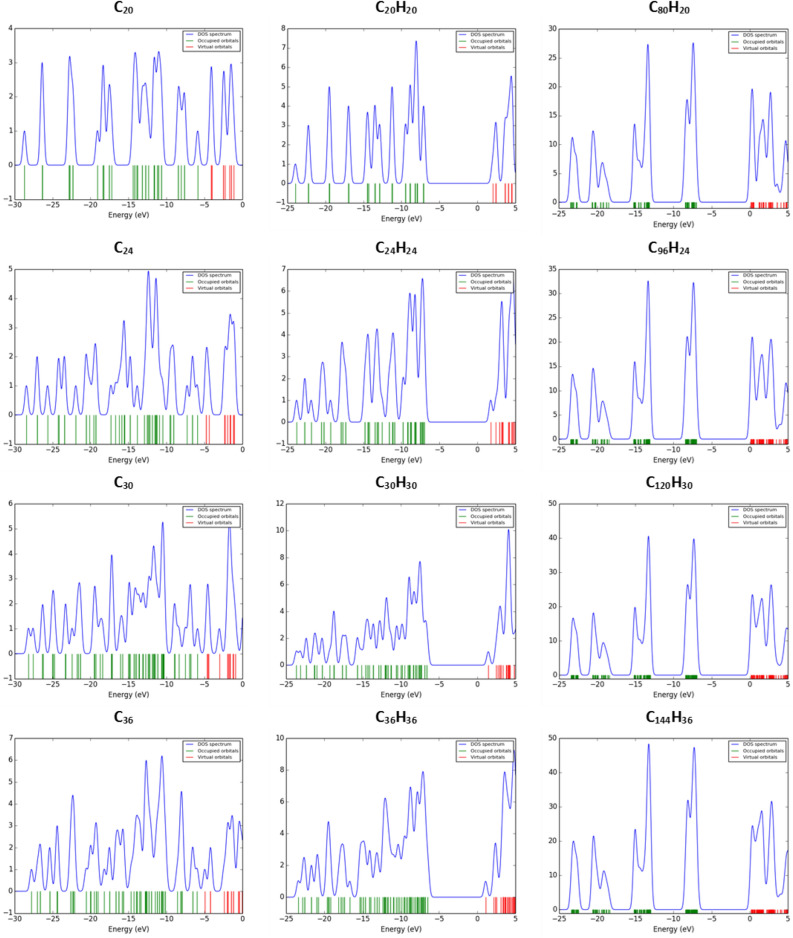
The density of the states’ diagrams in the fullerene, fullerane, and fulleryne structures, respectively. The density of states diagrams for the investigated nanocages are obtained using GussSumm software. Fullerenes have a larger electron affinity due to their negative LUMO levels, but fulleranes do not tend to capture electrons, due to a positive LUMO level. By considering the DOS of fullerynes, we see that their reactivity is between fullerenes and fulleranes, and unlike these two groups, as their size grows, their reactivity decreases.

Now, by considering fullerynes properties, we see that their reactivity is between fullerenes and fulleranes, and unlike these two groups, as their dimensions grow, their reactivity decreases. The density of the states (DOS) diagrams also confirms the distinction between the three groups of fullerenes, fulleranes, and fullerynes. The DOS diagrams for the investigated nanocages are obtained using GussSumm software^33^. The DOS plots are presented in Fig. 9. Fullerenes have a larger electron affinity due to their negative LUMO levels, but fulleranes do not tend to capture electrons due to a positive LUMO level. Although, the electron demand of the fullerynes is negative like that of the fulleranes, but not so strong, because its negative LUMO level is close to zero.

We use Natural Bond Orbital (NBO) analysis to explain the difference in DOS plots for the introduced fullerynes. There are two major peaks in the fullerynes DOS plots, unlike the fullerenes and fulleranes plots. NBO analysis shows that the peak between − 5 and − 10 eV is related to *sp* orbital hybridization. This orbital is related to the triple carbon bond added to the edges. There are two pi bonds in this orbital, each has a bond energy of about − 8 eV. As a result, the presence of two pi bonds at each edge of the fulleryne structures, causes the number of states to accumulate within this region.

The other major peak of the fulleryne plots is indirectly related to add two carbons to the edges of the fullerane structures again. Because it doubles the number of single carbon bonds with *sp*^3^ orbital hybridization. Therefore, the number of states related to *sp*^3^ orbital hybridization for fullerynes will be more than fullerenes and fulleranes.

### Optoelectrical properties

Another reason to categorize fulleryne as a new class of possible carbon nanocages is due to their distinct optoelectronic properties. Optical gap (OG), which is often different from HOMO–LUMO gap, is defined as the first singlet nonzero allowed optical transition.

**Figure 10:**
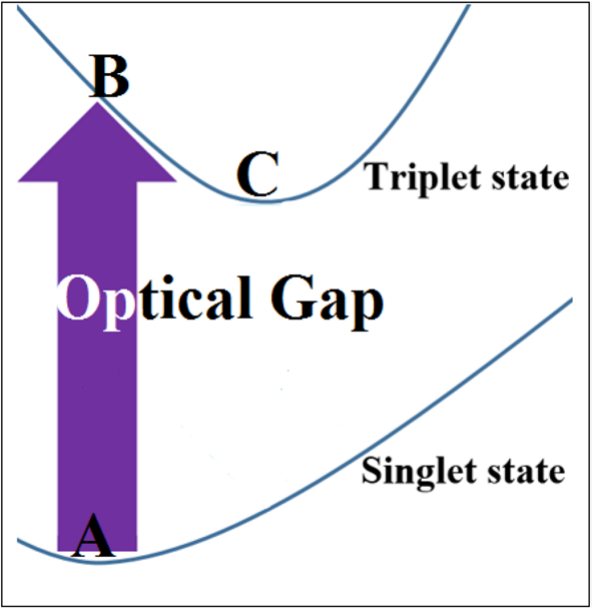
As shown in the figure, by calculating the total energy of particles, in singlet and triplet states at their optimized and ground states geometries, the optical gap gaps can be calculated.

According to $\Delta S$ = 0, optical transition rule, the first triplet excited state is optically inactive, therefore, the lowest excitation energy transfers the ground state, point A in Fig. 10, into the first singlet excited state, point B in Fig. 10, with the creation of an exciton or an electron–hole pair. Electrons with opposite spins experience larger repulsive Coulomb interaction. Therefore, singlet excited state being in higher energy level than the first triplet excited state, which may lead to a relaxation from the singlet excitation state to the triplet state (point C). OG is the difference energy between points A and B and is given as:

10 $$\Delta {E}_{OG}={E}_{B}-{E}_{A}.$$

The DFT-calculated OGs presented in Table 4, are the difference between the ground- and the first triplet excited-states^34–38^.

**Table 4:** Optical gap of the fullerynes and the corresponding fulleranes and fullerenes (in units eV).

Nanocages	Total energy		Optical gap
	Ground	Excited	
C_80_H_20_	− 83,206.76	− 83,201.24	5.52
C_96_H_24_	− 99,848.07	− 99,841.74	6.33
C_120_H_30_	− 124,810.09	− 124,804.51	5.58
C_144_H_36_	− 149,772.02	− 149,765.33	6.69
C_20_H_20_	− 21,062.94	− 21,054.80	8.14
C_24_H_24_	− 25,274.61	− 25,266.81	7.8
C_30_H_30_	− 31,592.11	− 31,584.92	7.19
C_36_H_36_	− 37,908.18	− 37,901.34	6.84
C_20_	− 20,714.29	− 20,713.74	0
C_24_	− 24,859.11	− 24,859.12	0
C_30_	− 31,080.30	− 31,080.07	0
C_36_	− 37,305.00	− 37,305.22	0

Considering the above data, we conclude once again that we are facing three different families of nanocages. As can be seen, the quantum confinement effect (QCE) is observed in the size of changes of the optical gap of the fulleranes^39^, but in the fullerynes, there is no regular downward change in the optical gap. The fullerenes gap is so small that it is not common in optical works and is more suitable for electronic applications^40^. One of the most important reasons for the differences in the properties of these three nanocages is the difference in the situation of their electrons. To clarify this, we investigate the linear correlation (***Y*** = A***X*** + B) of the structural gaps with their dimensions using the model of a particle in the box^41^.

11 $$\Delta {\text{E}} = \, \left\{ {{\text{const}}} \right\}{\text{/m}}^{*} \cdot {\text{L}}^{2} . $$

where we set the ΔE equivalent to the HOMO–LUMO gap and obtain 1/L^2^ from the values given in Table 1. Finally, we obtained for fullerene, ***Y*** = 157.57***X*** − 0.47, for fullerane, ***Y*** = 586.99***X*** + 4.96, and for fulleryne, ***Y*** = 266.99***X*** + 6.78, as a linear relation. We conclude that the effective mass of the electron is different in the three groups of fullerenes, fulleranes, and fullerynes. Since the slope of the line of relation to the fullerene is lower than the others, the heaviest effective mass is related to its electron. So, according to Fig. 8, we expect more stokes shift for the fullerane.

## Application

Fulleryne's chemical stability makes it suitable for storing and transporting some ions or gases at the nanoscale. Although fulleranes also exhibit higher chemical stability than fullerynes, but pentagon or hexagon of the fullerane structures cannot pass ions and molecules. In this subsection of the report, we have examined the results of the interaction of the fullerene structure with lithium ion. Using the following relationship, we can find the absorption energy (E_abs_) between ions and structures such as cages^42,43^.

12 $$ {\text{E}}_{{{\text{abs}}}} = {\text{ E}}_{{{\text{ion}}}} + {\text{E}}_{{{\text{cage}}}} - {\text{E}}_{{{\text{total}}}} . $$

The following table (Table 5) shows the results of the calculations of the absorption energy between the fulleryne cage (C_80_H_20_) with the atom and the lithium-ion in the center positions of the cage and its face. Note that, X@C_80_H_20_ means the particle is in the center of the cage, and X_C_80_H_20_ for the particle in the face of the cage.

**Table 5:** Table of energy, X@C_80_H_20_ means the particle is in the center of the cage, and X_C_80_H_20_ for the particle in the face of the cage (in units eV).

Cage + X	E_X_	E_Cage_	E_total_	E_abs_
Li@C_80_H_20_	− 203.84	− 83,209.81	− 83,413.63	− 0.02
Li_C_80_H_20_	− 203.84	− 83,209.81	− 83,413.34	− 0.31
Li^+^@C_80_H_20_	− 198.24	− 83,209.81	− 83,409.52	1.47
Li^+^_C_80_H_20_	− 198.24	− 83,209.81	− 83,410.55	2.50
H_2_@C_80_H_20_	− 31.43	− 83,209.81	− 83,241.24	− 0.00
H_2__C_80_H_20_	− 31.43	− 83,209.81	− 83,241.07	− 0.17

Our calculations show that the lithium atom in the center of the cage has negative and negligible absorption energy, and it gets the closer to the cage surface, its energy becomes bigger, and the more negative. As a result, if the absorbed lithium ion is converted to a lithium atom, it leaves the fulleryne cage.

**Figure 11:**
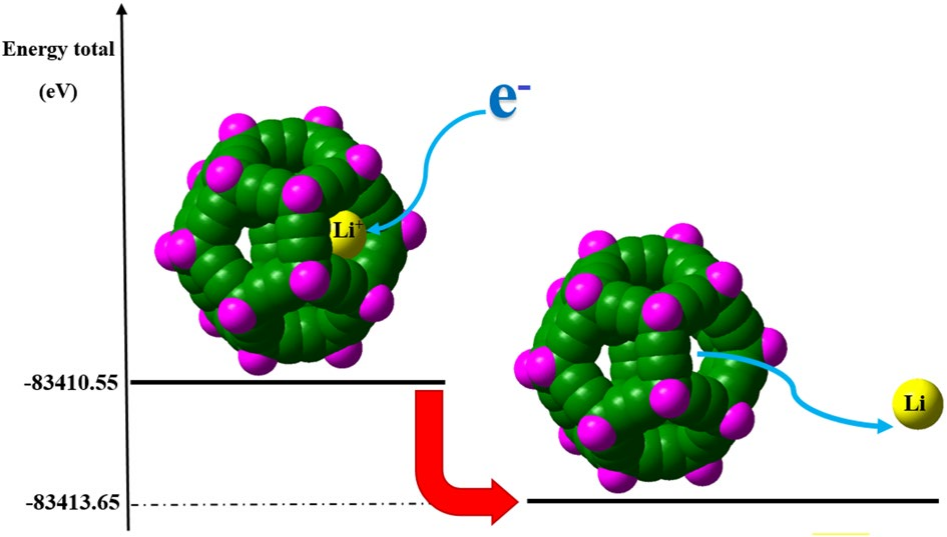
A schematic of the lithium atom inside the C_80_H_20_ fulleryne cage. Our calculations shows that the lithium atom, like a hydrogen molecule, can move freely in the fulleryne cage, while the lithium ion is bound to the fulleryne cage, especially in its face. This feature makes the fulleryne cages susceptible to use as cathode/anode electrodes in lithium-ion batteries.

MD calculations shows that fulleryne cages can serve as a nanoscale reservoir for hydrogen gas^44^, to find out how the ions are released into the fullerene cage; we compared their absorption energy with the hydrogen absorption energy in the cage, so that we can see the values in the table. Our calculations show that the lithium atom, like a hydrogen molecule, can move freely in the fulleryne cage, while the lithium ion is bound to the fulleryne cage, especially in its face^45^. This feature makes the fulleryne cage susceptible to use as a cathode/anode electrode in lithium-ion batteries. A schematic of the process of lithium-ion reduction and releasing lithium atom from the fulleryne cage is shown in Fig. 11.

According to the above figure and Table 4, it can be noticed that by oxidation of lithium atom, the energy level of the system increases (Discharge the battery).

## Summary

In this work, based on density functional theory, we have introduced a new branch of carbon nanocages; it is best to call them fulleryne, in the style of IUPAC, because of the triple bonds that exist in its structure. Examination of electrical, structural, and optical properties shows that the fullerynes fall into a category independent of known carbon cages.

Chemical stability of fullerynes makes them suitable for storing and transporting some ions or gases at the nanoscale. Besides, they can be proposed as cathode/anode electrodes in lithium-ion batteries.

## Method

**Figure 12:**
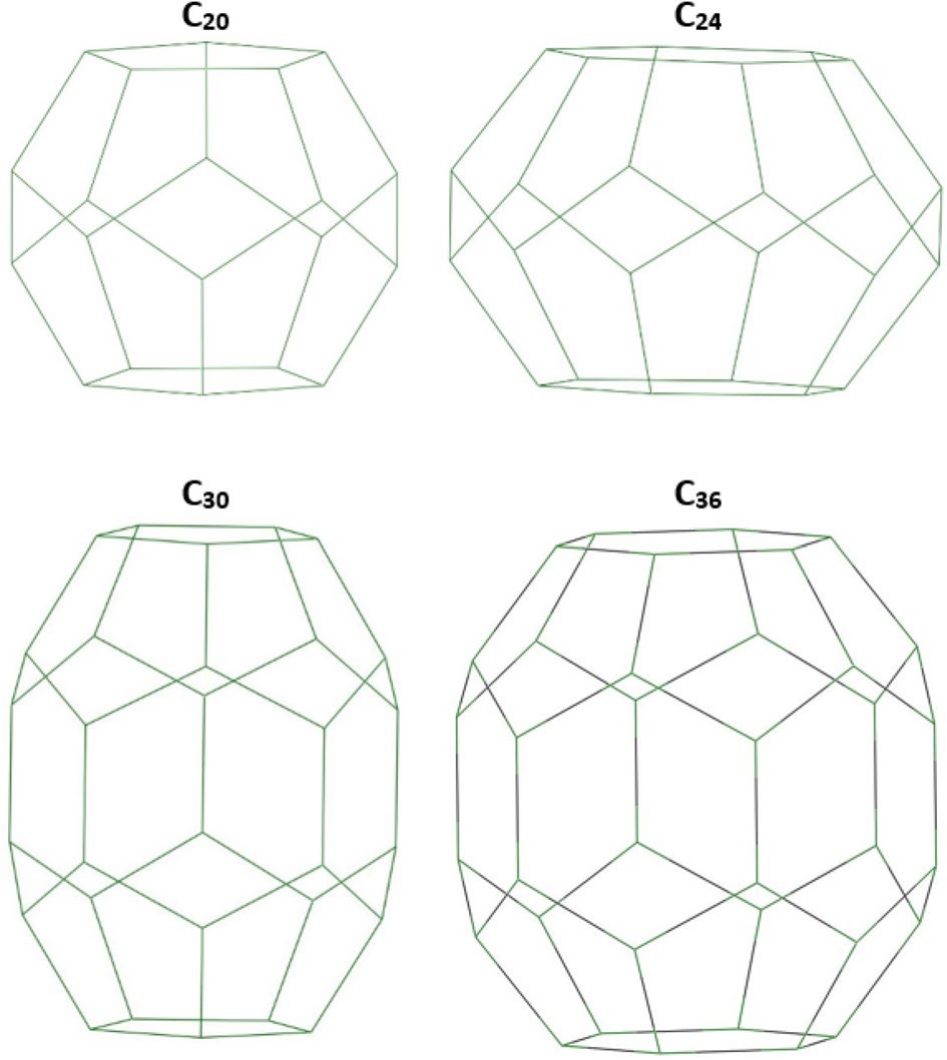
The geometry of the fullerenes, the C_20_, C_24_, C_30_ and C_36_ structures, are shown in the figure. The C_20_ is the smallest stable fullerene. The smallest fullerene has twelve pentagons in its structure, no hexagons. Fullerenes are carbon cages that their structural geometry consists of pentagons and hexagons. They have 12 pentagons, but the number of hexagons can be changed.

We used four fullerenes and four fulleranes to start our study. Consider the structures shown in Fig. 12. The following structures belong to four fullerene classes, by saturating the following structures with hydrogen, their corresponding fullerane structures emerge. Fullerenes are carbon cages that their structural geometry consists of pentagons and hexagons. Fullerenes have 12 pentagons in their structure, but the number of hexagons can change. Half of the carbon number in the fullerene cage minus 10 represents the number of hexagons of the fullerenes. The C_20_ is the smallest stable fullerene. The smallest fullerene has twelve pentagons in its structure and no hexagons in the geometry.

We added two carbon atoms on each edge of the above structures, and we got four new structures. We must first determine that these four new structures are stable. We did this by calculating the infrared frequency of the optimized structures. Finally, we identify the structural, electrochemical and optoelectrical properties of the new cages and compare their properties with fullerenes and fulleranes.

The computation of the total energy, the optimum structures, and frequencies of the vibrations to check stability, has been performed using density functional theory. We used the B3LYP hybrid functional, which includes three parameters of Beck's correlation, and also includes Li, Yang, and Par electron exchange, though it consists of a portion of exchange from the Hartree–Fock (HF) method, too^46,47^.

Elementary DFT method, underestimates the bandgap of the material, because it exaggeratedly predicts the density of occupied orbitals in a wide area, on the other hand, the HF method, gives localized unoccupied orbitals, so it overrates the bandgap^48^. Therefore, the results of the hybrid functional are more consistent with the experimental results.

In our calculations, for describing the shapes of the orbitals, we used the lanl2dz basis set^49–54^. This basis set only imports valence electrons into the computation, so reduces computation time by freezing the inner electron shells. Our calculations are performed by the Gaussian 98 package^55^.

## Data Availability

All data generated for this study are included in the article.
